# HDAC8 Inhibition Reduces Lesional Iba-1+ Cell Infiltration after Spinal Cord Injury without Effects on Functional Recovery

**DOI:** 10.3390/ijms21124539

**Published:** 2020-06-25

**Authors:** Sven Hendrix, Selien Sanchez, Elissia Ventriglia, Stefanie Lemmens

**Affiliations:** Department of Immunology, Biomedical Research Institute, Hasselt University, 3590 Diepenbeek, Belgium; seliensanchez@hotmail.com (S.S.); elissia.ventriglia@hotmail.com (E.V.); stefanie.lemmens@uhasselt.be (S.L.)

**Keywords:** HDAC inhibition, PCI-34051, valproic acid

## Abstract

Pan-histone deacetylase (HDAC) inhibition with valproic acid (VPA) has beneficial effects after spinal cord injury (SCI), although with side effects. We focused on specific HDAC8 inhibition, because it is known to reduce anti-inflammatory mediators produced by macrophages (Mφ). We hypothesized that HDAC8 inhibition improves functional recovery after SCI by reducing pro-inflammatory classically activated Mφ. Specific HDAC8 inhibition with PCI-34051 reduced the numbers of perilesional Mφ as measured by histological analyses, but did not improve functional recovery (Basso Mouse Scale). We could not reproduce the published improvement of functional recovery described in contusion SCI models using VPA in our T-cut hemisection SCI model. The presence of spared fibers might be the underlying reason for the conflicting data in different SCI models.

## 1. Introduction

Spinal cord injury (SCI) is a severe traumatic central nervous system (CNS) disorder for which no regenerative therapy is available. Currently, the standard of care consists of immunosuppressant drugs. However, these drugs offer little benefit and may cause detrimental side effects [1,2]. Therefore, new therapeutic strategies using more elaborate immunomodulation are desperately needed [1]. The pathophysiology of SCI can be divided into several phases [2], including the primary injury phase caused by the mechanical insult and a secondary injury phase initiated by a major neuroinflammatory response, neuronal cell death, edema, and the production of free radicals [3]. Monocyte-derived macrophages are important players in this neuroinflammatory response. They are fast reactors that produce several neurotrophic factors and cytokines and influence scar formation [4]. After SCI, macrophages are exposed to a variety of stimuli. Their phenotype changes quickly in vivo not only in the injured spinal cord, but also in other changing micro-environments [5,6,7]. Generally, they are situated between two spectral ends of functional activation: classically activated macrophages (M1) that secrete pro-inflammatory molecules contributing to deleterious effects and alternatively activated macrophages (M2) that secrete anti-inflammatory mediators as well as neurotrophic factors [8,9]. This division oversimplifies the functional diversity of macrophages in vivo, although it provides a useful concept to study macrophage function. Therefore, for pragmatic reasons, throughout this manuscript, these subtypes will be described as M1 and M2 macrophages, respectively [4,7,10,11,12]. M1 macrophages are dominantly present after SCI and exert detrimental effects, for example, by attacking dystrophic axons, while M2 macrophages are beneficial, promoting neurite outgrowth and reducing pro-inflammatory cytokines, although they are only transiently present. Their plasticity capabilities along with these specific properties make macrophages a promising target after SCI [10,13,14].

Histone deacetylase (HDAC) inhibitors are portrayed as promising therapeutics for neurodegenerative disorders because of their neuroprotective and anti-inflammatory effects [15,16]. For example, in vitro, broad HDAC inhibitors reduce pro-inflammatory markers in glial cultures and, in vivo, broad class I HDAC inhibition, using scriptaid, protected against white matter injury in a mouse model for traumatic brain injury (TBI) [17,18]. Furthermore, several neurodegenerative disorders are accompanied by a decreased histone acetylation [19,20,21]. Histone acetylation is regulated by histone acetyltransferases (HATs) and histone deacetylases. The balance kept between these enzymes determines the degree of gene expression. HATs regulate the addition of acetyl groups, while HDACs mediate the removal of acetyl groups from histone lysine residues of their target proteins. Typically, increased lysine acetylation leads to gene expression by loosening the chromatin condensation and reduced lysine acetylation leads to a more condense chromatin state, and thus attenuation of gene expression. However, HDACs do not only target histone acetylation. They play a much broader role by modulating other enzymes and proteins, thus they are involved in a broad range of biological activities including inflammatory gene expression and neuronal regeneration [19,20,21,22,23,24,25,26]. Previous studies have reported beneficial effects of HDAC inhibitors on the macrophage phenotype and in various models of CNS trauma and degeneration such as SCI, TBI, and stroke [21,26]. HDAC3 inhibition has been demonstrated to promote alternative activation of macrophages in vitro [27]. Valproic acid (VPA), a broad class I HDAC inhibitor, is known as a treatment for epilepsy and bipolar disorders. Several studies have demonstrated that VPA exerts neurotrophic, anti-inflammatory, and anti-apoptotic effects after SCI, and that it improves functional recovery in contusion and compression SCI models [20,21,28,29,30]. Although VPA has a confirmed safety and tolerability profile, the success of clinical studies in CNS disorders was limited and considerable side effects such as hepatotoxicity were observed [20,25,26,30,31]. These might be owing to the non-specificity of this broad-spectrum HDAC inhibitor. For that reason, a more specific HDAC inhibitor might overcome these drawbacks [19,25,26]. VPA targets all class I HDACs, namely, HDAC1, 2, 3, and 8. Therefore, it remains to be determined which HDAC is responsible for the beneficial effects shown using VPA in SCI. HDAC3 was previously studied by our group as a possible target; however, this revealed that HDAC3 inhibition has no effect on functional recovery in a T-cut hemisection mouse model of SCI [27]. As HDAC1 and HDAC2 are involved in various essential biological processes such as proliferation, cell survival, and apoptosis and HDAC1 has been shown to exert anti-inflammatory effects, inhibiting these enzymes could disbalance cell homeostasis and could even result in pro-inflammatory effects [24,32,33]. In this study, we focussed on HDAC8 for several reasons. HDAC8 inhibition reduced matrix metalloproteinase 9 (MMP-9) expression in lipopolysaccharide (LPS)-stimulated THP-1 monocytes [34,35]. This is important because MMP-9 is known to exert detrimental effects after SCI. Noble et al. found MMP-9 null mice to have reduced blood–spinal cord barrier (BSCB) disruption, and consequently decreased neutrophil infiltration after SCI [36]. Hence, blocking MMP-9 via HDAC8 inhibition may reduce BSCB disruption and immune cell infiltration. In addition, HDAC8 inhibition reduces the expression of interleukin (IL)-1β and IL-6 in these LPS-stimulated THP-1 monocytes.

Therefore, we hypothesize that HDAC8 inhibition modulates the macrophage phenotype to reduce the pro-inflammatory macrophages and, in this way, makes the environment after SCI more permissive for regeneration to improve functional recovery after SCI. Selected M1 and M2 markers were investigated in vitro on the protein level to determine the effects of the specific HDAC8 inhibitor PCI-34051 compared with the effects of VPA as a positive control. VPA was selected as a positive control because of its previously shown neurotrophic and anti-inflammatory effects next to its beneficial effects on functional recovery in contusion and compression SCI models [30,37]. Furthermore, these inhibitors were administered after T-cut hemisection SCI in mice and the Basso Mouse Scale (BMS) was used to determine whether they could improve functional recovery. We demonstrate that PCI-34051 has no effect on the macrophage phenotype in vitro, whereas VPA increases NO production. In vivo, we could not reproduce the published beneficial effect of VPA. PCI-34051 reduced Iba-1+ cell infiltration, but did not affect functional recovery after SCI. The results from this study underline the value of comparing different experimental set-ups and using several animal models to test the efficacy of a new potential treatment for SCI.

## 2. Results

### 2.1. PCI-34051 Has No Effects on Macrophage Phenotype, whereas VPA Significantly Increases NO2^−^ Production after LPS Stimulation In Vitro

**Figure 1:**
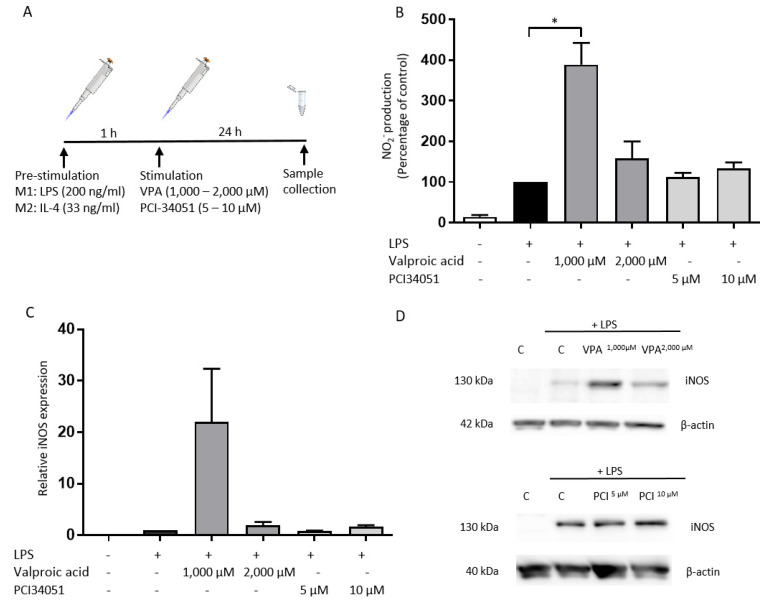
PCI-34051 has no effect on NO2^-^ production or iNOS expression, whereas VPA significantly increases NO2^−^ production after LPS stimulation. (**A**) BMDMs were stimulated with LPS for 1 h and then treated with VPA at 1000 µM and 2000 µM as a control and with PCI-34051 at 5 µM and 10 µM for 24 h. This experimental set-up was applied for all in vitro experiments. (**B**) Culture medium was collected to analyze the NO2^−^ production via a Griess assay. VPA (1000 µM) significantly increased NO2^−^ production after LPS stimulation. PCI-34051 does not affect nitrite production after LPS stimulation. (**C**,**D**) Total protein lysates were analyzed using Western blot analysis to examine the iNOS protein expression. VPA and PCI-34051 had no effect on iNOS production upon LPS stimulation. Representative blots are shown in d. Data are represented as relative values compared with control + LPS ± standard error of the mean (SEM); * *p* < 0.05; *n* = 3–4 independent experiments; BMDMs: bone marrow-derived macrophages; LPS: lipopolysaccharide; NO2^−^: nitrite; iNOS: nitric oxide synthase; C: vehicle control; VPA: valproic acid; PCI: PCI-34051.

Previous reports showed that several HDAC inhibitors can modulate macrophage responses [34,35]. Consequently, we tested the effects of the HDAC inhibitors VPA and PCI-34051 (HDAC8 inhibitor) on the macrophage phenotype in vitro. The macrophages were pre-stimulated 1 h with either LPS or IL-4, whereafter they were treated with the HDAC inhibitors for 24 h (Figure 1A).

The potency of these HDAC inhibitors was confirmed by testing their effects on histone 3 acetylation (Figure S1).

Next, the effects of PCI-34051 on protein expression of M1 and M2 markers were evaluated. VPA was tested as a control. The effects of PCI-34051 on the protein expression of iNOS and the production of NO2^−^ by M1 macrophages were investigated. PCI-34051 had no effect on NO2^−^ production, nor on iNOS expression (Figure 1B–D). NO2^−^ production was significantly upregulated after treatment with 1000 µM VPA (Figure 1B).

**Figure 2:**
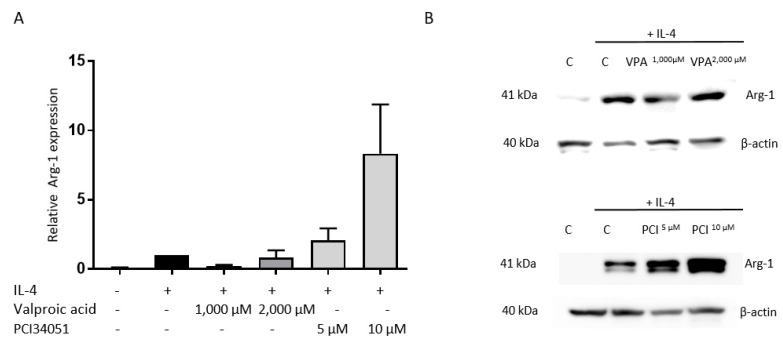
PCI-34051 and VPA have no effect on Arg-1 expression. BMDMs were stimulated with IL-4. After 1 h, these cells were treated with VPA at 1000 µM or 2000 µM as a control or with PCI-34051 at 5 µM or 10 µM for 24 h. (**A**) VPA and PCI-34051 have no effect on Arg-1 expression upon IL-4 stimulation. (**B**) Representative blots are displayed. Data represent percentages relative to control + IL-4 ± SEM; *n* = 3–4 independent experiments; BMDMs: bone marrow-derived macrophages; IL-4: interleukin 4; Arg-1: arginase-1; C: vehicle control; VPA: valproic acid; PCI: PCI-34051.

Additionally, the expression of the well-established M2 marker Arg-1 was examined [38]. Both inhibitors had no effect on Arg-1 expression (Figure 2).

### 2.2. PCI-34051 Reduces Iba-1^+^ Cell Infiltration whithout Effects on Functional Recovery, whereas VPA Has No Effects on Histopathological or Functional Recovery after SCI

**Figure 3:**
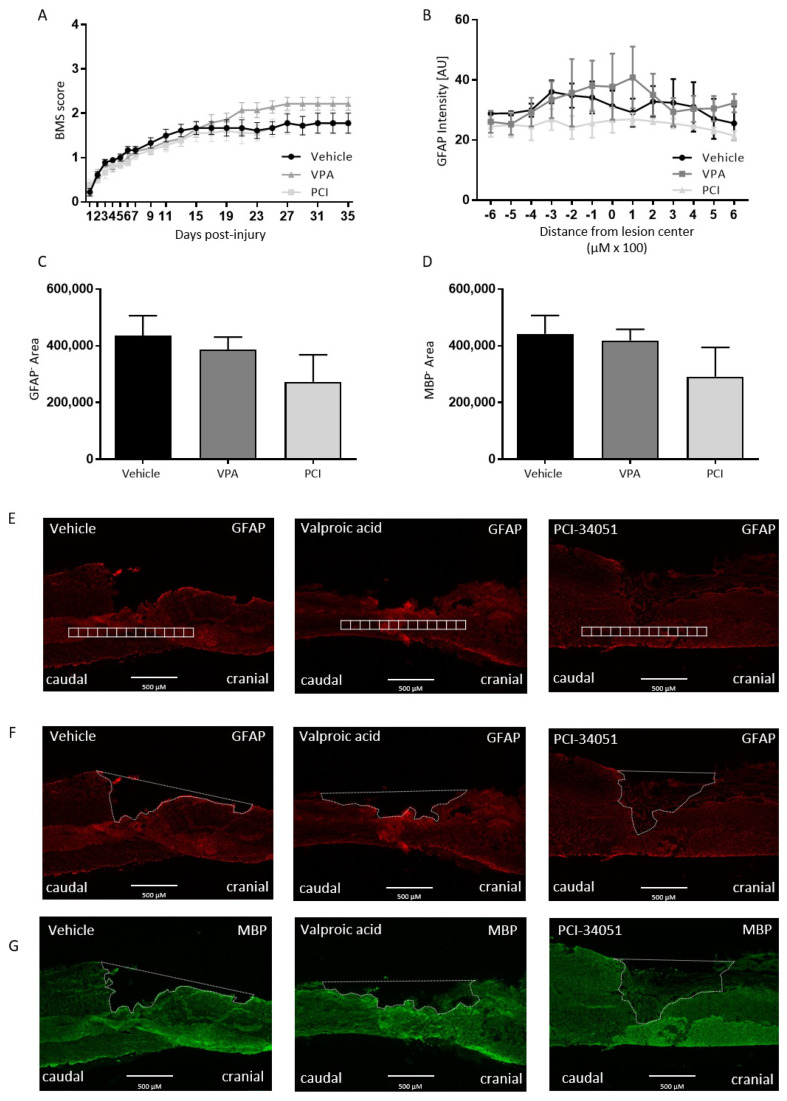
PCI-34051 and VPA have no effect on functional recovery, lesion size, or demyelinated area after SCI. BALB/c mice were subjected to a T-cut hemisection SCI. For the first 5 days, the mice were injected i.p. with VPA (250 mg/kg), PCI-34051 (20 mg/kg), or vehicle. (**A**) Recovery of hindlimb motor function was determined using the Basso Mouse Scale (BMS). Treatment with PCI-34051 has no effect on functional recovery after SCI. In addition, VPA showed no effect on functional recovery. Sections were labelled for GFAP (lesion size, astrogliosis) and MBP (demyelinated area). (**B**–**D**) No changes were observed for lesion size, astrogliosis, and demyelinated area when VPA or PCI-34051 were administered. (**E**–**G**) Representative images show the method of quantification: GFAP expression was quantified by intensity analysis within rectangular areas of 100 µm × 100 µm, extending 600 µm cranial to 600 µm caudal from the lesion epicentre. GFAP-area and MBP-area are delineated to evaluate the lesion size and demyelinated area. Data are represented as means ± SEM, *n* = 7–9 mice/group for BMS and *n* = 3 mice/group for the histological analyses. SCI: spinal cord injury; BMS: Basso mouse scale; GFAP: glial fibrillary protein; MBP: myelin basic protein; VPA: valproic acid; PCI: PCI-34051.

To test our hypothesis whether HDAC8 inhibition can improve functional recovery after SCI, PCI-34051 was tested in vivo after T-cut hemisection SCI. Again, VPA was included as positive control. The effect of VPA and PCI-34051 on functional recovery after SCI was investigated using the BMS. Strikingly, the results showed no effect of VPA or PCI-34051 on functional recovery after T-cut hemisection SCI (Figure 3).

**Figure 4:**
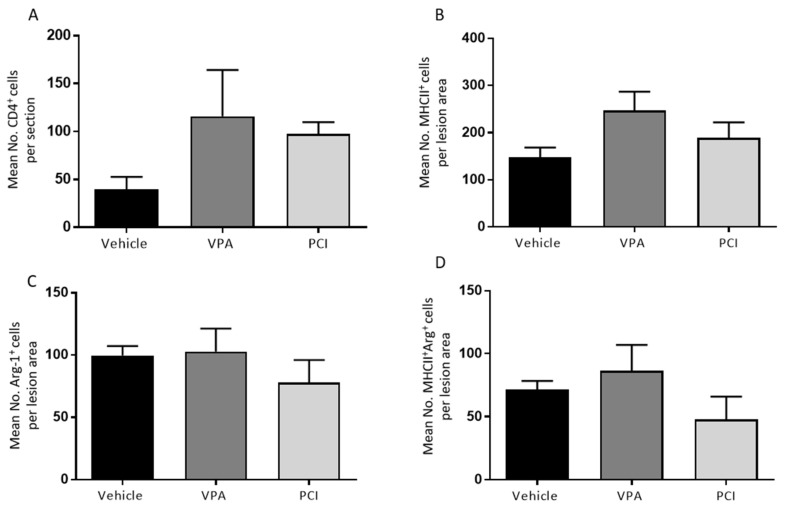
PCI34051 and VPA have no effect on CD4^+^, MHCII^+^, and Arg-1^+^ cell numbers after SCI. Spinal cord sections were labelled for CD4^+^ cells (T helper cells, **A**), MHCII^+^ cells (classically activated macrophages/microglia, **B**), and Arg-1^+^ cells (alternatively activated macrophages/microglia, **C**). (**A**) The number of CD4^+^ cells was counted in complete spinal cord sections. (**B**–**D**) MHCII^+^, Arg-1^+^, and MHCII^+^/Arg-1^+^ cells were counted at the lesion area. Data are represented as means ± SEM, *n* = 3–4 mice/group. ** *p* < 0.01. Arg-1: arginase-1; CD4: cluster of differentiation 4; MHCII: major histocompatibility complex 2; PCI: PCI-34051; SCI: spinal cord injury; VPA: valproic acid.

On the histological level, no differences were found compared with the control group when looking at lesion size, demyelinated area, astrogliosis (Figure 3), and cluster of differentiation 4 (CD4^+^) cell infiltration (Figure 4A).

**Figure 5:**
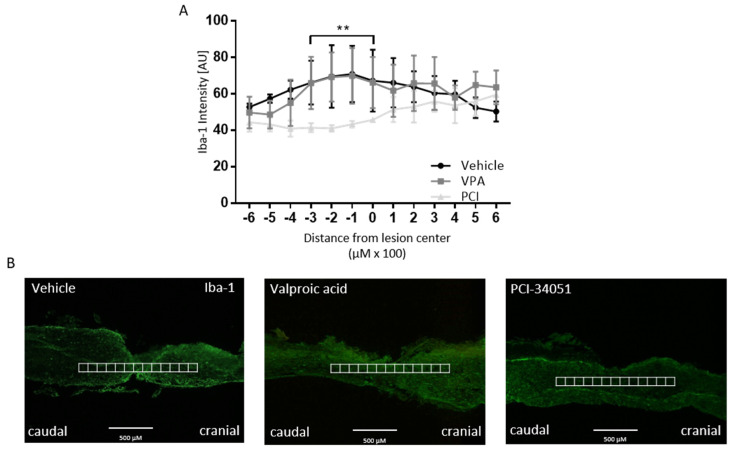
PCI-34051 reduces Iba-1^+^ cell infiltration after SCI. (**A**) PCI-34051 reduces Iba-1^+^ cell infiltration. VPA has no effect on Iba-1^+^ cell infiltration. (**B**) Representative images display the method of quantification: Iba-1 expression was quantified by intensity analysis within rectangular areas of 100 µm × 100 µm, extending 600 µm cranial to 600 µm caudal from the lesion epicenter. Data are represented as means ± SEM, *n* = 3–4 mice/group. ** *p* < 0.01. SCI: spinal cord injury; Iba-1: ionized calcium-binding adapter molecule 1; VPA: valproic acid; PCI: PCI-34051.

The effect of PCI-34051 on macrophage phenotype after SCI was determined because of the previously shown anti-inflammatory effects of HDAC8 inhibition [35]. However, PCI-34051 and VPA had no effect on the number of MHCII+ and Arg-1^+^ cells (Figure 4B–D). PCI-34051 did reduce the presence of Iba-1^+^ cells (Figure 5).

## 3. Discussion

In the present study, we investigated the effect of the specific HDAC8 inhibitor PCI-34051 in vivo in our T-cut hemisection SCI mouse model. We used VPA as a positive control to investigate whether the previously shown beneficial effects might be owing to targeting HDAC8. In addition, previous reports showed that several HDAC inhibitors can modulate macrophage responses. More specifically, HDAC8 inhibition may reduce pro-inflammatory mediators such as IL-1β, IL-6, and TNF-α in monocytes and macrophages [34,35]. Therefore, we hypothesized that the positive outcome of VPA in vivo in previous studies could be owing to a reduced M1 macrophage response mediated by HDAC8 inhibition. Consequently, we first tested the effects of these HDAC inhibitors on the macrophage phenotype in vitro. However, PCI-34051 had no effect on NO2^-^ production, nor on iNOS expression, although NO2^−^ production was significantly upregulated after treatment with 1000 µM VPA. Additionally, the expression of the well-established M2 marker Arg-1 was examined [38]. Both inhibitors had no effect on Arg-1 expression. These findings indicated that PCI-34051 has no effects on the macrophage phenotype in vitro, whereas VPA increased NO2^−^ production in primary macrophages. This is in contrast with previous studies indicating that specific HDAC8 inhibition may affect the phenotype of macrophages. Specifically, the HDAC8 inhibitor ITF2357 reduced the mRNA expression and production of IL-1β, IL-6, and TNF-α by LPS-stimulated peripheral blood mononuclear cells [39]. They did not address the effects on NO2^−^ production and iNOS protein expression. The effects of VPA are also in contrast to previous studies where reduced NO2^−^ production in BMDMs was found after combined treatment with VPA and LPS [28,29]. Guo et al. used the macrophage-like RAW264.7 cell line polarized towards the M1 phenotype via TNF-α and showed reduced NO production as well as iNOS expression upon VPA treatment [28,29]. This is in contrast to the BMDMs and LPS stimulation that we used, which could have caused the differences in effects. In addition, Serrat et al. used M-CSF to differentiate bone marrow cells towards macrophages, followed by M-CSF deprivation for 16–18 h before starting treatments [29]. We used L929 conditioned medium, which contains M-CSF and was kept in the culture medium during the experiments. M-CSF is important for the differentiation and maintenance of macrophages and, therefore, the M-CSF depletion might explain the differences in expression levels because M-CSF deprivation may have considerable effects on the macrophage phenotype [8]. The effect of VPA on NO production is only seen when treating the BMDMs with 1000 µM and not when they are treated with 2000 µM. A possible explanation is that the higher dose is out of range to produce effects on the inflammatory response. Previous studies using other HDAC inhibitors, SAHA and ITF2357, have demonstrated that the effects of HDAC inhibition are strongly dose-dependent. For example, doses that modulate inflammatory effects are much lower than doses leading to anticancer effects such as reduced proliferation and cell cycle arrest [40,41,42].

Previously, we have shown that the specific HDAC3 inhibitor RGFP966 increased Arg-1 expression upon IL-4 stimulation. These results indicate that an additional increase in Arg-1 expression is possible and that it is not yet at maximum level when only stimulated with IL-4 [27]. There is a trend indicating increased Arg-1 expression after treatment with 10 µM PCI-34051, although, owing to high variation, this trend does not reach statistical significance. Previous studies have shown effects of PCI-34051 on mitochondrial cell death genes and IL-1β expression in lower concentrations (20 nM and 100 nM) on toxin-induced resistant RAW264.7 macrophages [43,44]. In addition, PCI-34051 was found to be neuroprotective in concentrations between 1 µM and 10 µM. Furthermore, PCI-34051 has been shown to be toxic in concentrations higher than 10 µM [45].

To test our hypothesis whether HDAC8 inhibition can improve functional recovery after SCI, PCI-34051 was tested in vivo after T-cut hemisection SCI. Again, VPA was included as positive control. The effect of VPA and PCI-34051 on functional recovery after SCI was investigated using the BMS. Strikingly, the results of showed no effect of PCI-34051 or VPA on the BMS. These data are not the result of variation of the hemisection mouse model because the accuracy has been verified and outliers have been excluded. On the histological level, no differences were found compared with the control group when looking at lesion size, demyelinated area, astrogliosis, and CD4^+^ cell infiltration or the macrophage phenotype, although PCI-34051 did reduce the presence of Iba-1^+^ cells.

Previous studies reported contradictory effects of VPA after SCI in both contusion and compression models in rats. These studies revealed that functional recovery is significantly increased after VPA treatment in these models. Furthermore, a reduced lesion size and microglia/macrophage infiltration was shown [20,21,30,46,47]. A possible explanation for this difference in outcome after VPA administration may be the different SCI models used. A T-cut hemisection injury model is a laceration model that results in very clean and standardized lesions, completely transsecting the dorsomedial and ventral corticospinal tract, ruling out spared fibers. In contrast, contusion injuries are characterized by possible sparing of axons, thus VPA may have had positive effects on the spared fibers in those experiments [48]. In vitro, it has been shown that VPA significantly increased neurite outgrowth of primary spinal cord neurons and increased the mRNA expression levels of neurotrophic factors such as BDNF and GDNF; hence, it is plausible that it induces outgrowth of spared fibers [20,30,47]. Furthermore, the T-cut hemisection injury results in a more severe lesion than the contusion and compression injuries used in the other studies. This difference in injury severity may be another reason for the conflicting findings described above. It may be argued that the T-cut hemisection model is too severe to allow any therapeutically induced recovery. However, we have shown in several studies during the last decade that genetic or pharmacological interventions can lead to substantial histological and functional recovery [49,50,51,52,53].

To our knowledge, we are the first to study HDAC8 inhibition in SCI. However, HDAC8 inhibition has been suggested in previous reports to be involved in macrophage polarization in vitro [20,34,35,39]. Therefore, we reasoned there might be an effect on the inflammatory cell infiltration or the macrophage phenotype after SCI. Indeed, the results showed a reduced amount of Iba-1^+^ cells after SCI. This reduction in microglia/macrophage presence may be seen as beneficial because of the pro-inflammatory responses they can initiate [54,55]. This may be linked to the decreasing effects of HDAC8 inhibition on MMP9 expression by phagocytes.

Jan et al. demonstrated that HDAC8 inhibition reduces MMP9 expression in THP-1 cells in vitro [35]. MMP9 is involved in blood–spinal cord barrier (BSCB) damage and increased permeability; hence, a reduction in MMP9 may lead to reduced BSCB permeability [56]. Thus, it is possible that less Iba-1^+^ cells migrated to the lesion because PCI-34051 induced less damage to BSCB by reducing MMP9 expression [30,56]. Moreover, only suppressing the phagocytes after SCI is clearly not enough to translate into an improved functional recovery. A decreased Iba-1^+^ cell population does not necessarily mean that the inflammatory reaction is less severe, because this depends on the phenotype of the microglia/macrophages. This was further investigated by quantifying the MHCII^+^ (M1), Arg-1^+^ (M2), and MHCII^+^Arg-1^+^ cells (activated M2) cells. However, there was no effect found of PCI-34051 on the macrophage phenotype after SCI.

In summary, we found that the specific HDAC8 inhibitor PCI-34051 and broad-acting HDAC inhibitor VPA have no effect on macrophage polarization in vitro. Although, VPA did increase NO2^−^ production by M1 macrophages. Furthermore, we demonstrated that PCI-34051 and VPA do not improve functional recovery after SCI, using the T-cut hemisection injury model. This is in contrast to previous studies, which showed that VPA improved functional recovery after contusion and compression SCI. This difference in outcome may be owing to the presence of spared fibers in the previous studies, which were performed using contusion/compression injury. The conflicting results in our more severe SCI model without spared fibers demonstrate the importance of comparing different animal models when evaluating the therapeutic potential of HDACs and their inhibitors.

## 4. Materials and Methods

### 4.1. Isolation and Polarization of Bone Marrow-Derived Macrophages

Bone marrow-derived macrophages (BMDMs) were derived from bone marrow isolated out of femurs and tibias of female Balb/c mice (Envigo, Cambridgeshire, UK) as previously described [27,38]. The BMDMs seeded at 0.25 × 106 cells/cm^2^ were pre-stimulated for 1 h either with LPS (200 ng/mL; EMD Millipore, Billerica, MA, USA) or IL-4 (33 ng/mL; Peprotech, Rocky Hill, CT, USA). Thereafter, the BMDMs were stimulated for 24 h with VPA (1000 µM or 2000 µM, Sigma-Aldrich, Overijse, Belgium) or PCI-34051 (5 µM or 10 µM; Selleckchem, Bioconnect, TE Huissen, The Netherlands) without washing off the pre-stimulation. Western blot and Griess assay were performed to determine the effects on macrophage phenotype, as previously described [27,39,40].

### 4.2. Griess Assay

To measure nitrite (NO2^−^ concentration), which is a measure of NO production and a characteristic of M1 polarisation, a Griess assay was performed on LPS-stimulated cell media. This assay was performed using the ‘Griess reagent system’ kit (Promega, Leiden, The Netherlands) according to the manufacturer’s instructions.

### 4.3. Western Blot Analysis

Total protein lysates from stimulated BMDMs were collected using sodium dodecyl sulfate (SDS) lysis buffer (2% (*w*/*v*) in 125 mM Tris). Protein concentrations were measured using Pierce BCA protein assay kit (Thermo Fisher Scientific, Merelbeke, Belgium) according to the manufacturer’s instructions and iMARK Microplate Reader (Bio-Rad Laboratories, Hercules, CA, USA). Protein samples (10 µg) were separated on 12% or 7.5% (for iNOS) SDS gels for 45 min at 200 V. Western blot was performed as previously described [27,52]. The primary antibodies used were as follows: rabbit anti-mouse acetylated histone 3 lysine 9 (1/2000; Cell signalling, Leiden, The Netherlands), rabbit anti-mouse acetylated histone 3 lysine 27 (1/1000; Cell signalling), goat anti-mouse arginase-1 (Arg-1; 1/1000; Santa Cruz Technologies, Dallas, TX, USA), mouse anti-mouse inducible nitric oxide synthase (iNOS; 1/500; Sigma-Aldrich), and mouse anti-mouse -actin (1/5000, Santa Cruz). The measured values were normalized to the level of beta-actin or total histone 3, as internal controls. To improve readability of the images, the contrast was enhanced (same % as beta-actin per blot) and the images were cut in the representative protein blots in Figure 1 and Figure 2.

### 4.4. MTT Assay

Naive BMDMs were seeded in a 96-well plate (Greiner, Diegem, Belgium) at a density of 50,000 cells/well. After 24 h, the medium was replaced by standard fresh culture medium containing different concentrations of VPA (1 µM, 10 µM, 100 µM, 1000 µM, and 2000 µM) and PCI-34051 (0.1 µM, 1 µM, 5 µM, and 10 µM). PCI-34051 has been shown to be toxic in concentrations higher than 10 µM [41]. After 72 h incubation, MTT assay was performed as previously described [42].

### 4.5. Experimental Spinal Cord Injury and HDAC Inhibitor Treatment

Experiments were performed using 10-week-old female Balb/c mice (Envigo). The animals were housed in groups under regular conditions (temperature- and humidity-controlled, 12 h light/dark cycle, and food and water ad libitum) in a conventional animal facility at Hasselt University. All experiments were performed according to international standards described in European Communities Council directive 2010/63 and were approved by the local ethical committee of Hasselt University (approval code: 201752A1) in 21 September 2017.

T-cut spinal cord hemisection injury was performed as previously described [27,39,42,43,44,45]. Briefly, mice were anesthetized with 3% isoflurane (IsofFlo, Abbot Animal Health, Belgium), and a partial laminectomy was performed at thoracic level 8 (T8). Complete transsection of the dorsomedial and ventral corticospinal tract was induced by bilateral dorsal T-cut hemisection using iridectomy scissors (Figure S2). The T-cut hemisection results in a complete paralysis of the hindlimbs. Muscles were sutured, and the back skin was closed with wound clips (Autoclip, Clay-Adams Co. Inc., New York, NY, USA). A glucose solution (20%) was administered i.p. to compensate blood loss during surgery. As postoperative pain treatment, buprenorphine (0.1 mg/kg bodyweight Temgesic, Val d’Hony Verdifarm, Esneux, Belgium), was administrated subcutaneously close to the lesion site. Mice were placed in a recovery chamber (33 °C) post-surgery. Investigators remained blinded to the treatment groups for the duration of the study. Bladders of the mice were manually emptied daily until the micturition reflex returned spontaneously. Mice were treated i.p. for 5 consecutive days, starting 6 h after SCI with either VPA (250 mg/kg), PCI-34051 (20 mg/kg), or vehicle containing 9 % vehicle solution (30 % PEG400, 5% propylene glycol 0.5% tween-80 in NaCl; Sigma-Aldrich). The dosage of VPA (450 mg/kg, 350 mg/kg, and 250 mg/kg) was tested in a pilot study and 250 mg/kg was selected based on the lowest percentage of mortality. Functional recovery in mice was measured using the BMS starting one day after injury (dpi) until day 35 by a trained investigator blinded to the experimental groups [46]. The BMS is a 10-point locomotor rating scale (9 = normal locomotion; 0 = complete hind limb paralysis). Mice were scored by one to two blinded investigators in an open field. During the first 8 days, mice were scored daily, and afterward, only every second day. The given scores are based on hind limb movements made in an open field during a 4 min interval. The analysis was done using the mean of the left and right hind limb scores for each animal. The accuracy of the mouse model was based on the morphology of the lesion after immunohistochemistry considering the criteria lesion depth and width (Figure 3F,G). Exclusion criteria are complete transsections and lesions covering the total width of the image area. The accuracy was also based on the BMS. Mice were excluded when they started with a BMS > 1 at 1 dpi or had a BMS of 0 at 35 dpi.

### 4.6. Immunohistochemistry and Quantitative Image Analysis

At 35 dpi, animals were sacrificed. Immunofluorescence staining and analysis were performed as previously described [27,39,44]. Briefly, demyelination, lesion size, inflammatory infiltrates, and gliosis were analysed. Therefore, the following primary antibodies were used: mouse anti-GFAP (1/500, Sigma-Aldrich), rat anti-MBP (1/250, EMD Millipore), rabbit anti-Iba-1 (1/350, Wako Chemicals GmbH, Neuss, Germany), and rat anti-CD4 (1/25, BD Biosciences, San Jose, CA, USA). To identify classically or alternatively activated macrophages, rat anti-MHC-II (1/200, Santa Cruz Technologies) and goat anti-Arg1 (1/50, Santa Cruz Technologies) were used, respectively. MHCII was chosen as a M1 marker because it is a notorious problem in macrophage research to find reliable M1 markers. In our previous study by Dooley et al., we showed single positive MHCII^+^ cells as well as double positive MHCII^+^Arg-1^+^ cells in the perilesional area [49]. On the basis of these findings, we interpret MHCII^+^ single positive cells to be activated M1 macrophages and double positive MHCII^+^Arg-1^+^ cells to be activated M2 macrophages. Next, the sections were incubated with corresponding secondary antibodies: goat anti-rat IgG Alexa Fluor 568 and 488 (1/250, Invitrogen, Carlsbad, CA, USA), goat anti-mouse Alexa Fluor 568 (1/250, Invitrogen), goat anti-rabbit Alexa Fluor 488 (1/250, Invitrogen), donkey anti-goat IgG Alexa Fluor 555 and 488 (1/400, Invitrogen, Carlsbad, CA, USA), rabbit anti-rat biotin (1/400, Dako, Diegem, Belgium), and streptavidin 488 (1/2000, Invitrogen).

Quantitative image analysis was performed using unmodified pictures in ImageJ open source software. MBP^-^ area was delineated to evaluate the demyelinated area. In the same way, GFAP staining was used to evaluate the lesion size by delineating the GFAP^-^ area. Iba-1 and GFAP expression was quantified by intensity analysis within rectangular areas of 100 µm × 100 µm, extending 600 µm cranial to 600 µm caudal from the lesion epicentre. CD4^+^ cells were counted in total spinal cord sections. Arg-1^+^ cells and MHCII^+^ cells were counted at the lesion area. This area was standardized and the same standardized area was used for all sections to count the positive cells. The analyses were done on 4–7 spinal cord sections per mouse representing the lesion area.

### 4.7. Statistical Analysis

The analyses were performed using D’Agistino and Pearson omnibus normality test to determine normal distribution (GraphPad Prism 7.0, La Jolla, CA, USA). To compare two groups, a non-parametric Mann–Whitney test was used. When comparing multiple groups, Kruskal–Wallis with Dunn’s multiple comparison was used. For functional recovery in vivo and histological analyses, a two-way analysis of variance (ANOVA) for repeated measurements with Bonferroni’s post hoc test for multiple comparisons was used. Data were reported as mean ± standard error of the mean (SEM) and differences were considered significant when *p* < 0.05.

## Supplementary Materials

Supplementary materials can be found at https://www.mdpi.com/1422-0067/21/12/4539/s1.

## Abbreviations

AC-H3	acetylation histone 3
Arg-1	arginase-1
BMDMs	bone marrow derived macrophages
BMS	Basso mouse scale
BSCB	brain spinal cord barrier
CD4	cluster of differentiation 4
GFAP	glial fibrillary protein
CNS	central nervous system
HAT	histone acetyl transferase
H3K9	histone 3 lysine 9
H3K27	histone 3 lysine 27
HDACs	histone deacetylases
IL-4	interleukin-4
Iba-1	ionized calcium-binding adapter molecule 1
iNOS	nitric oxide synthase
LPS	lipopolysaccharide
MBP	myelin basic protein
MHCII	major histocompatibility complex 2
MMP-9	matrix metalloproteinase-9
PCI	PCI-34051
SCI	spinal cord injury
TBI	traumatic brain injury
VPA	valproic acid
