# Supplementary material for: HDAC8 Inhibition Reduces Lesional Iba-1+ Cell Infiltration after Spinal Cord Injury without Effects on Functional Recovery
Source: Int J Mol Sci. 2020 Jun 25;21(12):4539. doi: 10.3390/ijms21124539 (PMC7352158; doi:10.3390/ijms21124539)
Supplement: Supplementary file 1 [file ijms-21-04539-s001.pdf]

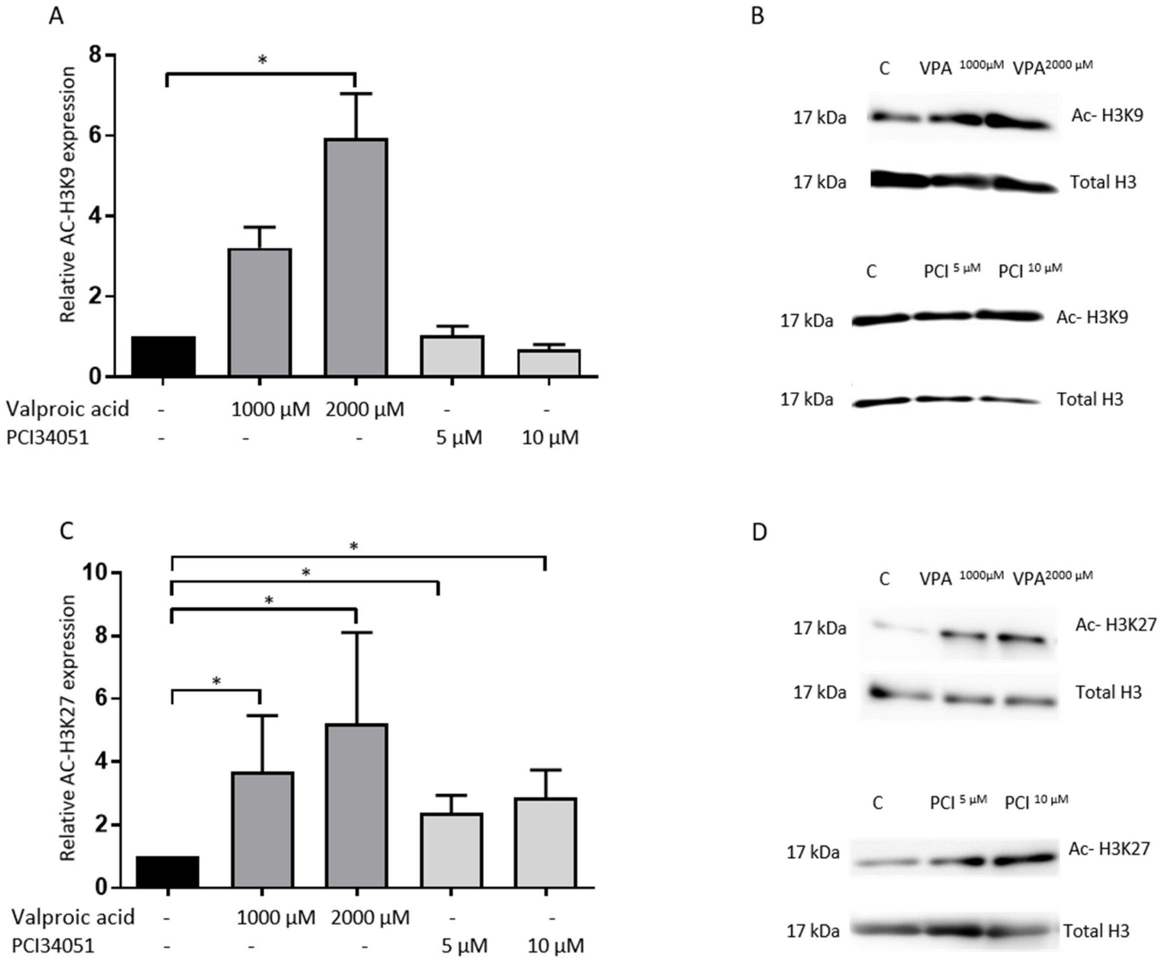

Figure S1: Valproic acid and PCI-34051 increase histone 3 acetylation at lysine residue 27. Bone marrow derived macrophages were treated with VPA or PCI-34051 in the indicated concentrations for 24 hours. Total protein lysates were analyzed using western blot to examine if these HDAC inhibitors could potently increase acetylation of histone 3. Total histone 3 was used as loading control. (a-b) First acetylation at H3K9 was tested. 2000 µM VPA significantly increased histone 3 acetylation whereas 5 µM and 10 µM PCI-34051 had no effect. (c-d) Therefore, acetylation at K27 was also analyzed VPA and PCI-34051 significantly increased H3K27 acetylation at both concentrations. Representative blots are shown. Data are represented as relative values compared to control ± SEM; *p < 0,05; n = 3-4 independent experiments; AC-H3: acetylation histone 3; C: vehicle control; VPA: Valproic acid; PCI: PCI-34051: H3K9: histone 3 lysine 9; H3K27: histone 3 lysine 27.

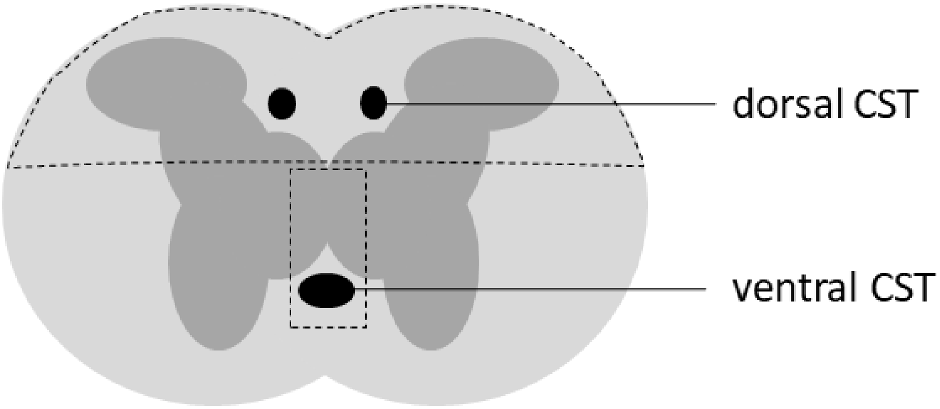

Figure S2: Schematic representation of the T-cut hemisection model. Complete transsection of the dorsal and ventral corticospinal tract (CST) was induced by bilateral dorsal T-cut hemisection using iridectomy scissors. The dotted lines indicate the cut areas. The first two cuts transsect the dorsal CST. The third cut transsects ventral fibers. Note: ventral CST fibers are not easily detectable. Therefore, there is sometimes a debate whether ventral CST fibers exist in all mouse lines. In our model, we have sporadically detected ventral CST fibers; therefore, we perform the T-cut hemisection to make sure that there are no spared ventral CST fibers left after transsection.
